# The New Scheme at King's College Hospital

**Published:** 1920-03-13

**Authors:** 


### CONTRIBUTIONS FROM PATIENTS.

### The New Scheme at King's College Hospital.

The interest aroused by the proposal to ask in-patients at the London Hospital to contribute ten shillings a week towards their maintenance invites attention to the scheme which has been adopted at King's College Hospital since October last.

The scheme concerns both in-patients, out-patients, and paying patients, and King's College Hospital claims to be the first to have adopted contributions on a recognised scale from the former. The in-patients or friends on their behalf are contributing according to their ability from half-a-crown to sixty shillings a week towards their maintenance. The out-patients, casualty, and early-morning dressing patients contribute sixpence per attendance; patients attending the massage departments, one shilling per attendance; and those who attend the dental department, half-a-crown. The financial result of the scheme cannot yet be known with certainty, but Mr. R. J. Coles and his committee estimate—or shall we say hope?—that the contributions of all classes of out-patients will produce £4,000 a year. On the same basis the contributions from in-patients should produce £6,000 a year. If we are not mistaken, the total £10,000 is the sum expected to be raised from the flat-rate contribution now being sought from in-patients at the London Hospital.

At King's College Hospital, moreover, provision was made some years ago for paying patients to be received in the private rooms by arrangement through one of the honorary physicians or surgeons. The number of beds available for paying patients was twenty-two, but since the military patients departed the Committee hopes to increase the number by placing at their disposal the rooms attached to the various wards. Once the recommendation of a member of the honorary staff has secured admission, the patient can choose between two types of rooms at different prices. The charge for rooms containing two beds is £4 4s. a week per patient, and £6 6s. a week for rooms with one bed only. These charges are for maintenance, and patients have to pay the fees of the physician, surgeon, and anæsthetist by arrangement with them. There is a great demand for these rooms, by which King's College Hospital is coming to the aid of the middle classes, or, as they have earned the right to be called, the new poor. The degree to which the funds of the hospital will benefit by this arrangement is not stated in the estimates at our disposal.

We believe that there is no eligible class for which King's College Hospital does not now directly cater. It trains not only medical students, but masseuses; and gives instruction in electrical and Swedish treatments. The public should remember that to treat the poor, to provide for the middle classes, to teach the student and the nurse, costs £80,000 a year. The assured income is £20,000.

Therefore, if those who are sick provide by the new plan a further £10,000 a year, those who are well have to combine to raise the remaining £50,000 required to square the deficit.

